# Evaluating the effectiveness of video-game based swallowing function training in patients with dysphagia: study protocol for a randomized controlled trial

**DOI:** 10.1186/s13063-023-07738-7

**Published:** 2023-11-16

**Authors:** Bohan Zhang, Cai Guo, Vivian Hui, Ka Po Wong, Yue Liu, Zihan Liu, Yanan Xu, Qian Xiao, Shu-Cheng Chen, Jing Qin

**Affiliations:** 1 Centre for Smart Health, School of Nursing, The Hong Kong Polytechnic University, Hung Hom, Kowloon, Hong Kong, China; 2 School of Computing and Information Engineering, Hanshan Normal University, Guangdong, China; 3 Department of Applied Social Sciences, The Hong Kong Polytechnic University, Hung Hom, Kowloon, Hong Kong, China; 4 Tiantan Xiaotangshan Rehabilitation Center, Beijing Xiaotangshan Hospital, Beijing, China; 5 School of Nursing, Capital Medical University, Beijing, China

**Keywords:** Dysphagia, Exercise, Rehabilitation, Game, Swallowing function, Randomized controlled trial, Protocol

## Abstract

**Background:**

Dysphagia can lead to serious complications such as aspiration and aspiration pneumonia, timely and effective rehabilitation training can improve the swallowing function of patients. However, the conventional rehabilitation training methods used in clinical settings have shortcomings such as poor adherence of patients. We present the study design of a randomized controlled trial that evaluated whether video-game based swallowing rehabilitation training can effectively improve swallowing in patients with dysphagia and whether it has additional benefits compared with conventional training methods to improve swallowing function and training compliance among patients with dysphagia.

**Methods:**

A randomized controlled trial with 4 weeks of intervention and 4 weeks of follow-up will be conducted in a rehabilitation center in Beijing, China. We will enroll 78 patients aged 18–80 years with dysphagia. Participants will be randomly assigned to the experimental group (video-game based swallowing function training) and the control group (conventional swallowing function training). All participants will receive 30 min of training per day, 5 times per week, for a total of 4 weeks. The primary outcome is swallowing function. Secondary outcomes include patients' quality of life, training compliance, and training satisfaction. Outcomes are assessed at baseline (pre-treatment), 4 weeks of treatment (post-treatment), and 8 weeks (follow-up), and the assessor is not aware of the participants’ grouping.

**Discussion:**

The protocol describes a new rehabilitation training method for dysphagia, which involves participant eligibility recruitment, recruitment strategies, and data analysis plan. The results of the study will inform the rehabilitation training and clinical care management of swallowing function in patients with dysphagia.

**Trial registration:**

ClinicalTrials.gov, NCT05978700. Registered on 28 July 2023.

## Administrative information

Note: the numbers in curly brackets in this protocol refer to SPIRIT checklist item numbers. The order of the items has been modified to group similar items (see http://www.equator-network.org/reporting-guidelines/spirit-2013-statement-defining-standard-protocol-items-for-clinical-trials/).

**Table:** 

Title {1}	Evaluating the effectiveness of video-game based swallowing function training in patients with dysphagia: study protocol for a randomized controlled trial
Trial registration {2a and 2b}.	ClinicalTrials.gov, NCT05978700. Registered on 28 July 2023. https://clinicaltrials.gov/study/NCT05978700
Protocol version {3}	Protocol version 1.0 (28 July 2023)
Funding {4}	N/A This research will apply for a funding in the future, and there is no funding to support at this time
Author details {5a}	B Zhang: Centre for Smart Health, School of Nursing, The Hong Kong Polytechnic University, Hung Hom, Kowloon, Hong Kong, China C Guo: Centre for Smart Health, School of Nursing, The Hong Kong Polytechnic University, Hung Hom, Kowloon, Hong Kong, China. School of Computing and Information Engineering, Hanshan Normal University, Guangdong, China V Hui: Centre for Smart Health, School of Nursing, The Hong Kong Polytechnic University, Hung Hom, Kowloon, Hong Kong, China KP Wong: Department of Applied Social Sciences, The Hong Kong Polytechnic University, Hung Hom, Kowloon, Hong Kong, China Y Liu: Tiantan Xiaotangshan Rehabilitation Center, Beijing Xiaotangshan Hospital, Beijing, China Z Liu: Tiantan Xiaotangshan Rehabilitation Center, Beijing Xiaotangshan Hospital, Beijing, China Y Xu: Tiantan Xiaotangshan Rehabilitation Center, Beijing Xiaotangshan Hospital, Beijing, China Q Xiao: School of Nursing, Capital Medical University, Beijing, China SC Chen: Centre for Smart Health, School of Nursing, The Hong Kong Polytechnic University, Hung Hom, Kowloon, Hong Kong, China J Qin: Centre for Smart Health, School of Nursing, The Hong Kong Polytechnic University, Hung Hom, Kowloon, Hong Kong, China
Name and contact information for the trial sponsor {5b}	Yue Liu, Tiantan Xiaotangshan Rehabilitation Center, Beijing Xiaotangshan Hospital, Beijing, China. 1131364506@qq.com
Role of sponsor {5c}	YL played no part in study design; collection, management, analysis, and interpretation of data; writing of the report; and the decision to submit the report for publication.

## Introduction

### Background and rationale {6a}

Dysphagia is a clinical phenomenon in which the jaw, lips, tongue, soft palate, pharynx, esophagus, and other organs are structurally and/or functionally impaired, preventing the safe and effective delivery of food from the mouth into the stomach [1]. Decreased muscle mass, reduced salivary secretion, and neurological disorders can lead to patients suffering from dysphagia [2]. A global survey reported that 26.2% of older adults, 8.1%-80% of stroke patients, and 11%-81% of Parkinson's patients had dysphagia [3]. Patients have increased complications such as aspiration, aspiration pneumonia, malnutrition, and psychological and social interaction disorders due to dysphagia, which leads to reduced immune response, prolonged patient length of stay in hospitals, increased patient mortality, and hospitalization costs [4].

Functional assessment and timely personalized swallowing training for patients with dysphagia could help improve the coordination of swallowing-related muscles, stimulate the central nervous system, expand the cortical sensory range, and promote the restoration and reconstruction of the swallowing reflex arc, which could effectively improve the swallowing level of patients [5]. However, the existing swallowing training was recently limited by space, the training mode was "one-to-one", and the training process was tedious [6–8]. Park et al. [9] used effortful swallowing training to train the muscles associated with swallowing during the oropharyngeal phase in patients with dysphagia. During the training, the therapist confirmed each patient's completion of an effortful swallow by visual observation and palpation [9]. As the number of patients with dysphagia increases, the "one-on-one" training model will eventually fail to meet the needs of the patients. Simultaneously, the boring and repetitive training leads to low patient compliance and adherence. Intermittent theta burst stimulation (iTBS) and repetitive transcranial magnetic stimulation (rTMS) require the assistance of professional staff and professional rehabilitation training equipment. The existing swallowing training equipments are mostly developed by professional staff, and rehabilitation training activities need to be carried out during hospitalization or in rehabilitation hospitals, making it difficult to achieve remote guidance and intelligence for swallowing function training, which makes patients suffer from untimely training and inappropriate rehabilitation [10, 11].

With the advent of the information technology age, more and more researchers are using video-game to help patients with their rehabilitation training. The researcher applies game thinking to the rehabilitation training environment, screens reasonable gamification intentions for training, creates a world that fits the patient, provides scenarios suitable for the experience of the patient, and enables the patient to engage in a variety of intuitive and natural real-time perceptual interactions such as visual, auditory, and tactile, thus making the rehabilitation process enjoyable and participatory [12, 13].

Video-game was currently rarely used for swallowing training in stroke patients, but mostly for physical and cognitive rehabilitation in stroke patients, and were proven to be effective [14, 15]. Park et al. (2019) designed a game-based chin tuck against resistance exercise to assist in rehabilitating patients with post-stroke dysphagia and found that patients had significantly higher training compliance and motivation scores, significantly lower exercise stamina, and muscle fatigue than the conventional training group [16]. However, Park et al. (2019) only performed the Chin tuck against resistance exercise, which did not include the entire swallowing training process needed for swallowing rehabilitation. Li et al. (2016) used a game-based biofeedback treatment that required patients to complete the training by swallowing hard and wearing a neck accelerometer that sensed the patient's swallowing threshold to enable interaction [17]. However, this study used acceleration curves to provide results on feedback and required lengthy training for medical staff to use. At this stage, there is still a lack of a video-game that are fully-trained and user-friendly for people with dysphagia, and a lack of high-quality, large sample size trials to verify the effectiveness of using video games for rehabilitation.

## Objectives {7}

This study aimed to investigate the effect of video-game on swallowing function in patients with dysphagia through a randomized controlled trial. It is hypothesized that video-game based intervention will improve swallowing function better in patients with dysphagia compared to conventional dysphagia training.

## Trial design {8}

This study is a single-center, single-blind, parallel randomized controlled trial (RCT) with a 4-week intervention and a 4-week follow-up. All phases are conducted according to the SPIRT reporting guidelines [18]. Treatments received will be randomly generated by R software in 1:1 numbers, randomly assigned by a postgraduate student not involved in the assessment and intervention, and placed in opaque closed envelopes. The intervention program will be based on the protocol of this study.

## Methods: participants, interventions and outcomes

### Study setting {9}

The study will be conducted at a rehabilitation center in Beijing, China. Ethical approval for the study has been obtained from the Institutional Review Board of the hospital (Approval No. LS20230720-1) and the study will be conducted with the consent of the hospital and department heads. Informed consent will be obtained from all participants before allocation.

### Eligibility criteria {10}

The inclusion criteria were: (1) diagnosis of dysphagia according to the Toronto Bedside Swallowing Screening Test (TOR‐BSST); (2) age between 18 and 80 years; (3) no significant cognitive impairment, able to execute instructions correctly, and with Mini-Mental State Examination (MMSE) score ≥ 24; (4) be able to use a computer screen with or without the assistance of others; (5) be able to understand and follow instructions; (6) life expectancy estimated by healthcare providers to be ≥ 12 months; and (7) informed consent to the study can be given by signing an informed consent form.

The exclusion criteria were: (1) dysphagia caused by structural lesions (eg, radiotherapy, previous extensive surgery of the head and neck region such as laryngectomy and cordectomy); (2) combined with serious heart, lung, liver, kidney diseases, and hematological disorders; (3) limb deficiency or paralysis, blindness in both eyes, severe visual impairments; (4) motion sickness or vestibular dysfunction; (5) history of epilepsy, malignancy or other neurological diseases; or (6) pregnancy or breastfeeding. Some of the exclusion criteria depended on the video-game device or were precautions designed to not harm participants after using video-game technology. Because of the need to use video games, there is concern that people over the age of 80 will not be able to use computers or view electronic screens, thus limiting participants under the age of 80.

### Who will take informed consent? {26a}

Based on the above criteria, patients with dysphagia who have been screened for eligibility to participate in this study will be invited to meet with the investigator to discuss the remaining questions and sign the informed consent form.

### Additional consent provisions for collection and use of participant data and biological specimens {26b}

If a patient withdraws consent, their data will be removed from the database and will not be used for analysis. In the case of study discontinuation, data may be retained if authorized by the patient. In such cases, primary and secondary outcomes will be assessed at the date of discontinuation.

## Interventions

### Explanation for the choice of comparators {6b}

We wish to observe the effect of video games on the rehabilitation of swallowing function. Therefore, the control group use the same components of the rehabilitation program without the video game effect. Both groups have the same duration of intervention.

### Intervention description {11a}

#### Instrumentation

A video-game based swallowing exercise was developed by our team, consisting of a computer screen, and a facial recognition device. To ensure the patient-centered design of video-game, we adopted the TURF (Task, User, Performance, and Function) unified usability framework [19]. In TURF, usability refers to "the usefulness, usability, and satisfaction of the target user in achieving the goals of the work domain by performing certain sequences of tasks". This framework was developed for healthcare settings and is often the basis for ensuring good usability in design. Participants will face a computer and a facial recognition device, which is constructed from a facial landmark detector in the MediaPipe Artificial Intelligence model library. It will generate the 3D positions of 468 facial feature points on the facial images, including information about cheeks, mouth, chin and other facial areas [20]. Through changes in the 3D position of facial feature points, changes in facial muscles and expressions of participants will be detected, and the computer obtains biofeedback through real-time game-controlled computation.

#### Intervention

**Fig. 1:**
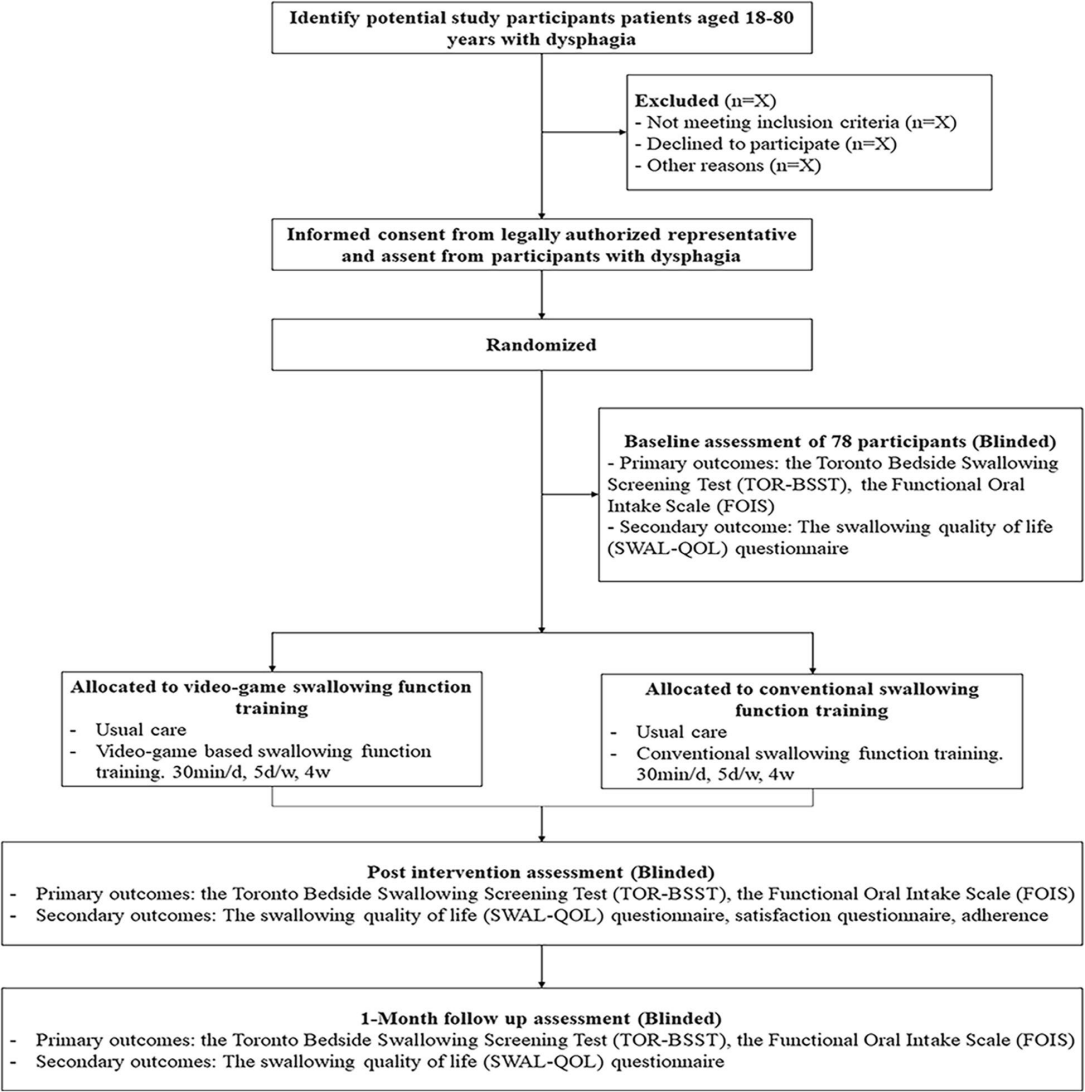
Study flow chart and procedure

The intervention was given once a day for 30 min per session, 5 times a week for 4 weeks. The experimental group received the video-game intervention and the control group received the conventional therapy intervention. Both groups received the usual care, including daily feeding education, position training, breathing control, improving cough technique, and thermal tactile stimulation. The researcher will participate in the patient's first three rehabilitation sessions to help guide the patient on rehabilitation methods, and the next 17 sessions will be done independently by the patient (Fig. 1).

**Fig. 2:**
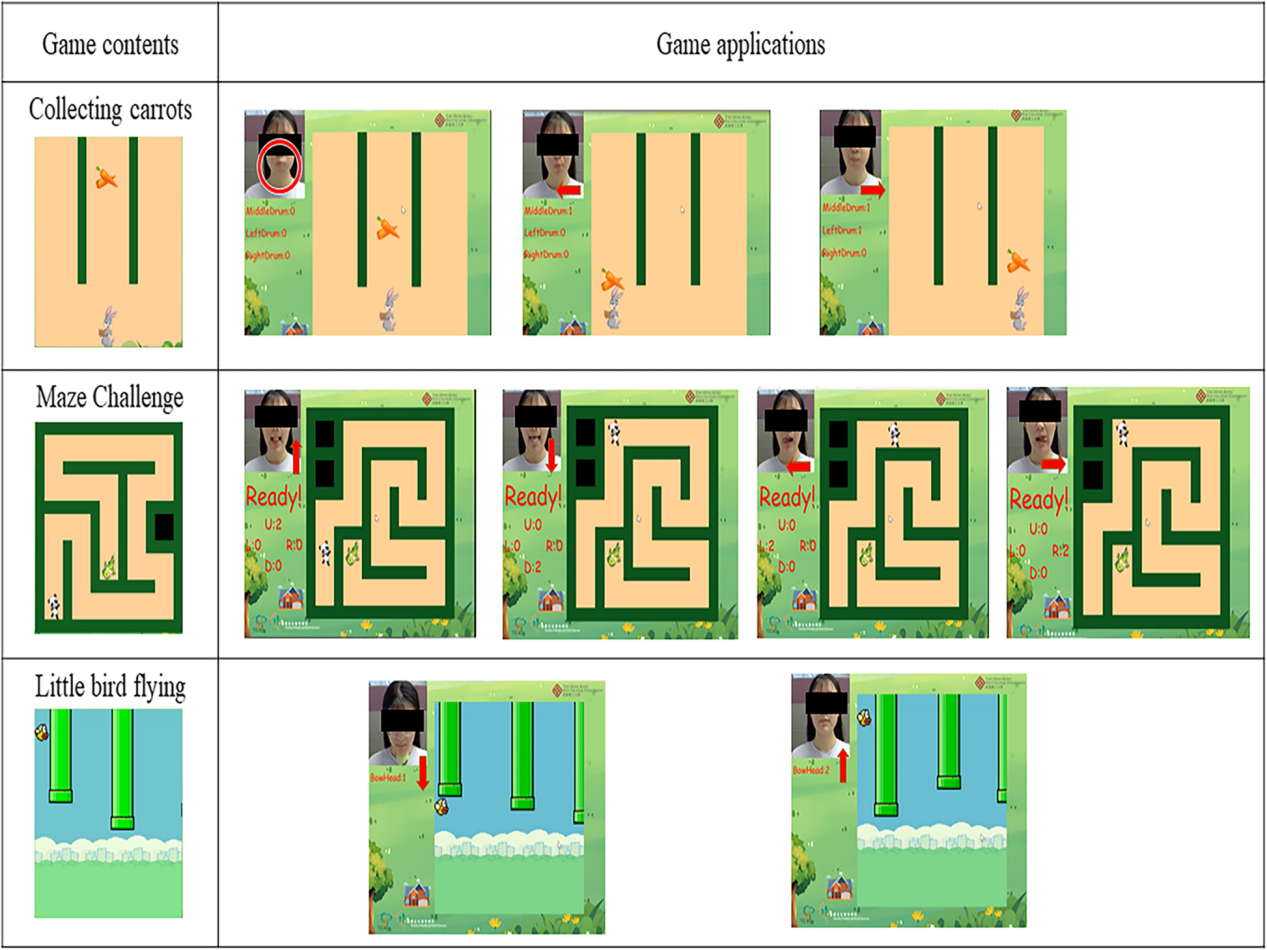
Game contents and applications of the video-game based swallowing function training. Each game application targets a specific movement of the swallowing training. The target movements are represented by red arrows and circle

The experimental group used video-game equipment for swallowing function training. Participants in the experimental group were asked to sit on a chair, facing the computer screen, and complete the video-game training process. The video game rehabilitation training contains a total of three games (Fig. 2).

Game One—Lip Exercise: the game “Collecting Carrots” appears on the computer screen. Participants move the character in the game by drumming cheeks, left drumming cheeks, and right drumming cheeks, hold the movements for 2–3 s. After the character in the game moves to the left and right to reach the designated place, the carrot will automatically fall into the back basket of the character in the game, participants need to complete drumming cheeks, left drumming cheeks, and right drumming cheeks 15 times, respectively.

Game Two—Tongue Exercises: the “Maze Challenge” game appears on the computer screen. The participant uses tongue movements to make the characters in the game move. The participant extends the tongue, tongue up, tongue down, tongue left, tongue right, and the game character in the labyrinth is activated, up, down, left, and right by the participant's tongue movements. When the participant sticks out his tongue once and holds it for 3 s, the game character will move one space in the corresponding direction. The patient can rest for 2 s before proceeding. The route out of the maze was designed based on the route of the movement program of the patients’ tongue, and the game character walked out of the labyrinth when the patient completed the tongue movement. Each movement repeats 15 times.

Game Three – Lower Jaw Exercise: the “Little bird flying” game appears on the computer screen. In this game, participants control the direction of the bird's flight by lowering their jaw. Participants use their chin to press the rubber ball, hold it for 2–3 s, and then lift it up and the bird will fly down and around the obstacle. Participants are required to go around the obstacle a total of 15 times.

The control group used the conventional swallowing function training. The lip exercise consisted of the following specific movements: opening mouth, closing mouth, drumming cheeks, left drumming cheeks, and right drumming cheeks; the tongue exercise consisted of the following specific movements: extending the tongue, tongue up, tongue down, tongue left, and tongue right. Each specific movement in the steps lasts 2-3 s, repeat 15 times and continue with the next movement. The lower jaw movement contains the following specific movements: keep the head as low as possible, and squeeze the rubber ball placed on the neck for 2–3 s, repeat 15 times.

### Criteria for discontinuing or modifying allocated interventions {11b}

Neither the measurements nor the game manipulation produces any significant harmful side effects, as the game does not have graphic depictions of violence, and any of our measurements are not invasive. Therefore, we do not anticipate the need to modify the intervention during the study.

Patients can withdraw from the study at any time without adverse consequences for the patient. The investigator can also terminate a patient's participation in this study if the patient is uncooperative and/or does not attend the study visits. Patient data collected to date will be included in the analysis. If too much data is lost, the patient will be replaced by a new patient. The study will be terminated early in the event of serious adverse events, such as aspiration or vomiting.

### Strategies to improve adherence to interventions {11c}

Video-game based swallowing rehabilitation programs are safe with multiple game components, so high compliance with the program is expected. Adherence is based on the actual completion of the patient's swallowing function training. The actual completion of the dysphagia training by patients, including the number of training sessions completed and the duration of the training, is objectively recorded by the researcher. In addition to this, participants will be asked to complete a daily training diary in which they record the training activities they perform and record the duration, intensity, and symptoms of discomfort.

### Relevant concomitant care permitted or prohibited during the trial {11d}

Patients are allowed to receive other necessary basic care, such as daily feeding education, position training, breathing control, and improving cough technique, and medications. Each group will be given the same duration and intensity of basic usual care, using standardized operating procedures, in order to reduce the effect of usual care on the effectiveness of the intervention. The progress and status of treatment in both groups will be monitored weekly to ensure that usual care is performed consistently.

### Provisions for post-trial care {30}

There is no anticipated harm and compensation for trial participation. Neither the measurements nor the game manipulation produces any significant harmful side effects, as the game does not have graphic depictions of violence, and any of our measurements are not invasive.

### Outcomes {12}

All assessments were completed by an experienced occupational therapist who was blind to the participants' group assignments. Assessments were conducted before the start of the intervention (pre-training), after 4 weeks of treatment (post-training), and at the 1-month follow-up (follow-up).

The primary outcome of this study was swallowing function, assessed using the Toronto Bedside Swallowing Screening Test (TOR-BSST) and the Functional Oral Intake Scale (FOIS). The Toronto Bedside Swallowing Screening Test (TOR‐BSST) is the best performing water swallow screening tool [21] with a sensitivity of 91.3%, a negative predictive value of 93.3% in the acute phase and 89.5% in the recovery phase [22]. TOR-BSST has shown to be more convenient and cheaper in bedside screening compared to the gold standard method of simultaneous videofluoroscopy (VFSS), which is an invasive assessment. Due to the limitations of the experimental site, we chose TOR-BSST as the assessment tool for dysphagia. FOIS consists of a 7-point scale, with level 1 indicating completely impaired oral intake and level 7 indicating complete oral intake regardless of food concentration or type [23].

Secondary outcomes in this study were quality of life, nutritional status, satisfaction and adherence. The swallowing quality of life (SWAL-QOL) questionnaire consists of 10 subscales and a symptom scale (14 items) to assess the severity of dysphagia symptoms. Scores range from 0 to 100, with lower scores indicating greater impairment of quality of life from dysphagia [24]. Body mass index (BMI) and Mini Nutritional Assessment Short Form (MNA-SF) were used to assess the nutritional status of the dysphagia patients. BMI is one of the most commonly used nutritional assessments among adults [25]. MNA-SF is commonly used to screen patients for malnutrition or risk of malnutrition with high sensitivity and specificity [26]. MNA-SF in combination with BMI showed higher accuracy [27]. The satisfaction questionnaire was designed based on the literatures. There are 15 items, involving 3 dimensions: training mode content setting, training mode format setting, and self-subjective feeling, and scored on a 5-point Likert scale. The higher the score, the more satisfied the patients were with the video-game training. Adherence was considered good if the patient could complete more than 80% of the training sessions of the corresponding training program; average if the patient could complete 50% to 80% of the training sessions; and poor if the patient could complete less than 50% of the training sessions. Patients are considered to have completed their training when they have completed more than 12 training sessions [28].

### Participant timeline {13}

The participant timeline was presented in Table 1. (Table 1 was placed at the end of the document text file).

**Table 1:**
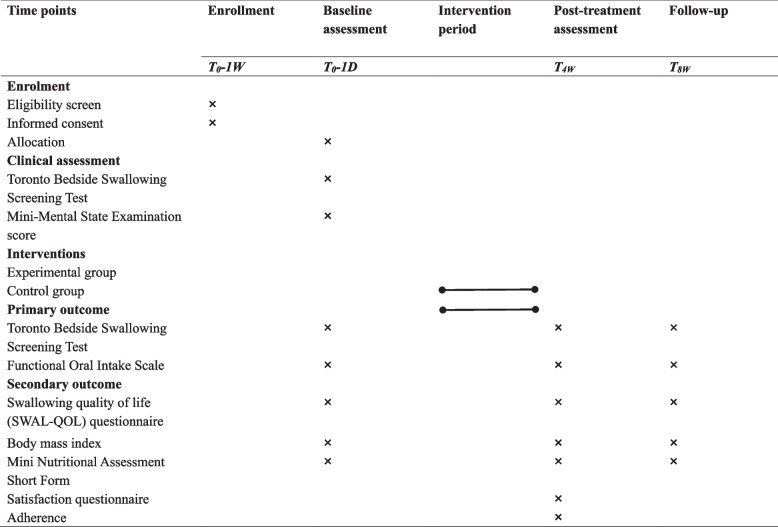
Time schedule of enrollment, interventions, and assessments

### Sample size {14}

The sample size was calculated from a previous study conducted by Park et al. [29] in dysphagia patients, which measured patient swallowing function on the Functional Oral Intake Scale (FOIS), with mean of 5.40 and 4.40, SDs of 1.70 and 0.80 for the intervention and control groups, respectively. For a significant level of 0.05 and power of 90%, 31 participants were estimated in each group (62 participants). Finally, to avoid loss of power due to potential dropout, the sample size was increased by 20% for each group, resulting in a total number of participants of *N* = 78. The sample size was estimated using G-Power 3.1 [30].

### Recruitment {15}

All patients with dysphagia who meet the inclusion criteria are eligible to participate. The study will recruit patients at the rehabilitation center by distributing posters and leaflets containing information about the study. We will collaborate with doctors and rehabilitation therapists, request their help in promoting publicizing the study after the clinic treatments. In addition to this, we will actively promote the study to patients in the wards.

## Assignment of interventions: allocation

### Sequence generation {16a}

To avoid selection bias, an independent statistician will use R software to generate a set of random numbers. The list of random numbers will consist of a set of numbers ranging from the number 1 to the number 78, and they will be randomly divided into intervention group and control group. During the randomization process, the R function "sample ()" will be used to ensure the same group size. The intervention group will be named "Group 1" and the control group will be named "Group 2".

### Concealment mechanism {16b}

A research assistant (a postgraduate nursing student), who will not be involved in any session of the study, will place the randomized numbers in an opaque envelope and number them sequentially, then randomly assign the participants.

### Implementation {16c}

After consent and baseline assessment, eligible participants will receive sequentially numbered envelopes and will be assigned to either the intervention or control group based on the numerical number inside the envelope.

## Assignment of interventions: blinding

### Who will be blinded {17a}

The evaluator will blind to the assignment of all participants, and will not participate in the application of the treatment or the statistical analyses. Due to the specific nature of video-game interventions, it is not possible to blind participants and therapists.

### Procedure for unblinding if needed {17b}

N/A: Only outcome evaluators in this study will be blinded, so unblinding will not occur.

## Data collection and management

### Plans for assessment and collection of outcomes {18a}

Each participant will be assessed individually three times, pre-intervention, post-intervention and follow-up. The assessments will focus on swallowing function, quality of life, nutritional level and satisfaction with video games. Data will be collected by the investigators on site using case report form (CRF). All data obtained during the study will be anonymized and stored in folders in a protected research server at the Hong Kong Polytechnic University. Only the research team is allowed to access the folder.

### Plans to promote participant retention and complete follow-up {18b}

During the recruitment process, participants will receive information about the study design and requirements. The researcher will explain to participants the benefits and implications of participating in the study and emphasize the importance of completing the study. Participants are permitted to withdraw from the study at any time during the study. Throughout the follow-up period, the researcher will monitor the participant's status at all times and contact the participant as necessary to complete the study. If participants withdraw from the study, efforts will be made to collect data on outcomes up to the time of withdrawal, with the participant's consent.

### Data management {19}

Participant data will be collected through the CRF. Protected folder will be backed up once a month. Informed consent and trial end date will be recorded in the patient dossier. All changes made to the raw data and all steps taken in the analysis will be recorded in the CRF and IBM SPSS. All study data, including patient information, will be archived for 10 years after the end of the study.

### Confidentiality {27}

All data in this study will be anonymized and strict confidentiality measures will be taken. Access to participant data will be restricted to authorized members of the research team and the Ethics Committee of The Hong Kong Polytechnic University. To enhance participant confidentiality, each participant will be provided with a unique trial identification number and his/her details will be securely stored in a protected database at The Hong Kong Polytechnic University. Access to the dataset will be strictly controlled and will only be granted to the research team and the sponsoring organization. It is important to note that participant identification details will not be reported in any publications or studies. In addition, the possibility of sharing anonymized trial data with other researchers for international prospective meta-analyses will be carefully considered, and appropriate measures will be taken to protect participant privacy and confidentiality, such as thoroughly de-identifying all direct identifiable information of participants before data sharing, and processing indirect identifiable information that may contain sensitive information, such as date of birth, by keeping only the year or age, in order to protect the privacy and confidentiality of participants.

### Plans for collection, laboratory evaluation and storage of biological specimens for genetic or molecular analysis in this trial/future use {33}

N/A: There are no biological samples in this study.

## Statistical methods

### Statistical methods for primary and secondary outcomes {20a}

An intention-to-treat analysis will be performed, and the last available measurement will be used when data were lost due to dropout. Statistical analysis will be performed using SPSS 29.0. Statistical significance of all outcome variables will be established at *P* < 0.05.

All continuous data will be tested for normality, and nonparametric tests will be used for non-normally distributed data. Data conforming to normal distribution will be expressed as mean ± standard deviation (SD), and data with non-normal distribution will be expressed as median (interquartile range).

Comparisons of continuous data of two groups at the same time will be analyzed using independent samples t tests or rank sum tests, and comparisons of two groups of rates will be performed using chi-square test or Fisher exact test [31].

Comparison of continuous data trends over time between different groups at different times, if data normality is present, we will perform a series of repeated measures analysis of variance (ANOVA) for each outcome measure to assess differences between time (4 weeks and 1 month post) and between study groups, as well as interactions between time and study groups. For these analyses, the assumptions of sphericity and homogeneity of variances are tested using Mauchly and Levene tests [32]. If the assumption of sphericity is violated, the Huynh–Feldt correction is applied [32].

If the normality assumption is not met, the linear mixed model will be used for analysis, the comparative analysis at each time was performed using the nonparametric statistical Mann–Whitney U test and its own control two-way comparison using the nonparametric statistical Kruskal–Wallis rank sum test [33].

### Interim analyses {21b}

N/A: There will not be interim analyses.

### Methods for additional analyses (e.g. subgroup analyses) {20b}

N/A: No subgroup analyses or adjusted analyses will be performed because they are not part of the planned analyses for this study. The primary analysis is sufficient to address the research question.

### Methods in analysis to handle protocol non-adherence and any statistical methods to handle missing data {20c}

An intention-to-treat analysis will be performed, and the last available measurement will be used when data were lost due to dropout. To address missing data, the multiple imputation method will be employed. For participants randomized to the intervention group who do not adhere to the intervention, a per-protocol analysis will also be performed to assess the impact of non-adherence on the study outcomes.

### Plans to give access to the full protocol, participant level-data and statistical code {31c}

The project has been made public in clinical trials. The datasets and statistical codes analyzed in this study and the complete study protocol are available from the corresponding author upon reasonable request.

## Oversight and monitoring

### Composition of the coordinating centre and trial steering committee {5d}

This is a study designed, implemented and coordinated by the Hong Kong Polytechnic University. Day-to-day support for the study is provided by the Principal Investigator. The Data Manager is responsible for organizing data collection and ensuring data quality. The study coordinators help with trial registration, collect informed consent, ensure follow-up according to protocol, and address participant concerns. The study team will meet every two weeks throughout the duration of the study. There will be no trial steering committee or stakeholder and public participation meetings. The Hong Kong Polytechnic University Ethics Committee will check the completeness of the study.

### Composition of the data monitoring committee, its role and reporting structure {21a}

Data monitoring committee (DMC) was not specified for this study. This decision was made on the basis that no serious adverse events would occur in this study.

### Adverse event reporting and harms {22}

Adverse events will be documented by the research coordinator. Minor adverse events such as fatigue will be recorded in the adverse event count form. A healthcare staff who is not involved in the study will accompany the participants during their training. Since the use of video games as an intervention method may cause discomfort such as visual fatigue in participants, the study will be stopped and observed immediately when participants experience any discomfort. If the discomfort is not relieved after half an hour, it will be immediately reported to the doctor in charge and relevant treatment will be given. Serious adverse events, such as fractures, and bleeding will be reported immediately to the Health Research Ethics Committee and discussed with the participant's doctor, who will provide medical follow-up if necessary. Participants may withdraw from the trial at any time.

### Frequency and plans for auditing trial conduct {23}

A project management team comprised of the Principal Investigator will meet monthly to review recruitment, project compliance and identify any issues. As the risk of intervention is low, the establishment of an independent data safety monitoring commissioner will not be considered.

### Plans for communicating important protocol amendments to relevant parties (e.g. trial participants, ethical committees) {25}

All substantive modifications to this study will go through a structured communication process. The Principal Investigator (PI) will first inform the sponsor of any proposed modifications. Subsequently, the PI will seek approval from the Ethics Committee and the competent authorities of The Hong Kong Polytechnic University. Once the modification is approved, the PI will immediately notify the participating centers for the modification and provide a copy of the revised proposal, which will be added to the Investigator Site File accordingly. Any deviation from the protocol will be recorded in detail using the breach report form to ensure transparency and accountability. In addition, the protocol in the Clinical Trial Registry will be updated in a timely manner to reflect approved revisions. This comprehensive approach to protocol revision aims to maintain the integrity of the study while keeping all interested parties informed.

### Dissemination plans {31a}

The results of this study will be fully disclosed in the international peer-reviewed journals. Both positive and negative results will be reported. A summary of the results will be presented to the school authorities.

## Patient public involvement

This study did not involve public and patient representatives in the study design. However, after the preliminary development of the video-game system was completed, a total of 19 participants, including health caregivers, patients with dysphagia, and their families, were involved to experience the game and interviewed, providing many informative suggestions for video-game system modifications.

## Discussion

Swallowing training combined with video games may be a more feasible and effective intervention strategy than using usual care alone to improve swallowing function in patients with dysphagia. However, there is no evidence for the role of video-game based swallowing function training. Therefore, this study aims to provide new insights into whether video-game based swallowing function training can be an effective way to enhance swallowing function in patients with dysphagia. This will be the first known longitudinal study to combine a complete swallowing training program with video-game.

Swallowing movements can be understood as nutritional intake and airway blocking movements. In healthy individuals, the food bolus ingested through the mouth is transported through the esophagus to the stomach [34]. There are four consecutive coordinated phases of swallowing: the oral preparation phase, the oral propulsion phase, the pharyngeal phase, and the esophageal phase. The peripheral muscles involved in swallowing movements include the masticatory muscles, facial muscles, suprahyoid muscles, soft palate muscles, pharyngeal muscles, infrahyoid muscles, and intrinsic lingual muscles [35]. In patients with neurological disorders, central nervous system disorders lead to dysfunction and incoordination of the oropharyngeal muscles, and thus patients are likely to have dysphagia [36]. Also, advanced age or weakness of the muscles of the face, palate, and pharynx may increase the odds in developing dysphagia among patients. Therefore, appropriate muscle rehabilitation is a common and effective way to improve the swallowing function of patients.

Video games have deep potential for patient rehabilitation, such as reducing neuromotor impairment [37], improving cognition and attention [38], reducing anxiety and depressive symptoms [39], or managing chronic conditions [40]. Gamification is a useful strategy to achieve effective completion of rehabilitation by combining it with certain therapeutic interventions [41], such as muscle training, and by inspiring patients with points or rewards to increase their participation and motivation. Some serious games or video games for the education of patients with dysphagia have shown positive results [42]. However, to our knowledge, there are no trials that have studied the evaluation of the effectiveness of video-game for rehabilitation in patients with dysphagia for motor purposes.

This randomized controlled trial will not only provide evidence for the immediate effects of swallowing rehabilitation, but also for the short-term effects of video-game by follow-up. This study will help to estimate the variance of primary and secondary outcome indicators, correlations between measurement times, and effects with time. With more accurate sample size calculations, this will help confirm the need for a larger definitive trial targeting dysphagia rehabilitation. It will also determine whether video-game based swallowing function training is a useful intervention strategy for patients with dysphagia.

Although the results of this study may have important implications in the field of dysphagia rehabilitation, there are some limitations. First, the use of video games for training of patients with dysphagia has some requirements for the patients' ability to use a computer or computer configuration. This may result in some families of people with dysphagia not having access to video games. We have kept the computer usage requirements as low as possible, so that patients can simply click on the software and select the game to begin training. Second, blinding participants, carers and people delivering the interventions is very difficult due to the intervention method. We will require participants not to discuss study details with each other and conduct pre-experimental training for implementers to strictly follow experimental protocol details for minimizing the risk of bias. Third, because of the limitations of the study site, we were not able to use the gold standard video fluoroscopic swallowing study (VFSS) as a tool to assess dysphagia. Instead, we used the Toronto Bedside Swallowing Screening Test, which has been proven to have high sensitivity and specificity. This would lead to some limitations in the reliability of the results. Fourth, since we used some questionnaires in the study, this may increase recall bias and Hawthorne effects in participants' questionnaires. We will strictly train questionnaire investigators to require standardized answers to participants' questions in order to reduce participants' self-consciousness.

This randomized controlled trial will examine the effect of video-game based swallowing training on swallowing function, swallowing-related quality of life, and adherence to rehabilitation training in patients with dysphagia. It may provide evidence on swallowing function training, clinical care management, and lifestyle interventions for patients with dysphagia.

## Trial status

The current protocol is version 1 of August 2023. The trial status is currently in the pre-recruitment phase. Recruitment is planned to start in October 2023. Recruitment and trial are expected to be completed by July 2024.

## Data Availability

The final trial data for this protocol can be supplied on request.

## Abbreviations

BMI: Body mass index
CRF: Case report form
DMC: Data monitoring committee
FOIS: Functional oral intake scale
MNA-SF: Mini nutritional assessment short form
RCT: Randomized controlled trial
SD: Standard deviation
SWAL-QOL: Swallowing quality of life
TOR-BSST: Toronto bedside swallowing screening test
TURF: Task, user, performance, and function
VFSS: Video fluoroscopic swallowing study
